# Health Risk Assessment of Metal(loid) Contamination in Raw Cow’s Milk from the Colombian Caribbean Region

**DOI:** 10.1007/s12011-025-04728-5

**Published:** 2025-08-07

**Authors:** Fabio Fuentes-Gandara, Jaime Barreto-Cañas, Siday Marrugo-Madrid, José Marrugo-Negrete, José Pinedo-Hernández, Sergi Díez

**Affiliations:** 1 Department of Natural and Exact Sciences, Universidad de la Costa, Barranquilla, Colombia; 2 Faculty of Basic Sciences, Universidad del Atlántico, Barranquilla, Colombia; 3 Faculty of Basic Sciences, Department of Chemistry, Water, Applied and Environmental Chemistry Group, University of Córdoba, Monteria, Colombia; 4 Environmental Chemistry Department, Institute of Environmental Assessment and Water Research, IDAEA-CSIC, E-08034 Barcelona, Spain

**Keywords:** Potentially toxic elements, Milk, Risk, Public health

## Abstract

Milk is a daily source of essential nutrients, but the presence of trace metals raises significant health concerns about the quality of dairy products. This study investigated the presence of and human health exposure to potentially toxic elements (PTEs) in raw cow milk produced in the Caribbean region of Colombia, during both dry and rainy seasons. A total of 100 samples were collected from farms in five zones and analysed for toxic metal(loid)s, including Pb, Cr, Hg and As. Metal(loid) concentrations were generally higher during the rainy season, except for Hg, which showed elevated concentrations during the dry season. During the rainy season, values increased by factors of 20, 44, 3 and 0.5 for Pb, Cr, As and Hg, respectively. The year-round median concentrations of Pb, Cr, As and Hg in milk were 2.23, 44, 2.93 and 0.18 µg/L, respectively. The hazard index (HI) was higher than 1 in both adults and children, indicating a significant health risk of exposure to metal(loid)s in the milk‐consuming population. Frequent milk consumption posed no public health concern for carcinogenic risk related to Pb and As across age groups. However, the estimated carcinogenic risk for Cr exceeded the USEPA guidance reference value for both children (1.7·10^−4^) and adults (4.8·10^−4^) indicating a potential public health concern across all age groups. In conclusion, it is essential not to underestimate the health risks associated with frequent milk consumption. Immediate action and stricter regulations on consumption are urgently needed to prevent potential public health emergencies.

**Supplementary Information:**

The online version contains supplementary material available at 10.1007/s12011-025-04728-5.

## Introduction

Milk is considered a superfood, rich in essential nutrients like casein for bones and tissue repair, fat-soluble vitamins (A, D, E and K) and minerals such as calcium and phosphorus, all vital for bone and teeth health [1, 2]. These products constitute a valuable source of dietary energy, high-quality proteins and fats, contributing significantly to the necessary intake of nutrients such as calcium, magnesium, selenium, riboflavin, vitamin B12, pantothenic acid, in at-risk populations, particularly children, pregnant women and the elderly [3, 4] due to the various disorders that can develop and manifest later in consumers, especially in children [5]. PTEs, encompassing both metals and metalloids (hereafter referred to as metal(loid)s), pose a significant environmental and public health concern due to their pronounced toxicity, even at low concentrations. For instance, chromium (Cr) is a recognized human carcinogen, linked to renal, hepatic, and respiratory damage [6]. Lead (Pb), in turn, induces a wide array of adverse human health effects, including enzymatic dysfunction, severe acute and chronic intoxications, delayed neurological development, exacerbation of Alzheimer’s disease and various types of cancer [7]. Regarding mercury (Hg), its established neurotoxicity and propensity for bioaccumulation in tissues can lead to profound cognitive and motor impairments [8]. Finally, arsenic (As) has been correlated with the development of skin, lung and bladder cancers, in addition to significant cardiovascular and neurological effects [9].

Studies indicate that metals and metalloids (hereafter referred to as metal(loid)s) in cow milk primarily originate from contaminant intake by animals, mainly through contaminated water and forage. These contaminants come from various sources, including industrial waste discharge, pesticide residues in agriculture, wastewater from urban and rural areas and natural processes like volcanic activity [10, 11]. Urban expansion intensifies contamination issues by reducing agricultural land and increasing reliance on foods from potentially contaminated areas [12]. Industrial and commercial activities contribute metal(loid)s to soil and water, affecting crop quality [13]. Combined with inadequate regulation, this raises exposure risks, especially for vulnerable populations, leading to long-term health issues such as neurological disorders and kidney diseases [14].

The concentrations of metal(loid)s in milk and its derivatives fluctuate based on the environmental contamination levels of the surroundings [15–17], although the packaging materials used during the production chain also influence it [18–21]. Milk, being a natural product consumed worldwide, had a global production (approximately 81% cow’s milk, 15% buffalo and 4% combined goat, sheep and camel milk) estimated at around 897 million tons in 2022, indicating a 0.7% increase compared to the previous year. Global milk production is projected to grow annually at a rate of 1.5% in the next decade, reaching 1039 million tons by 2032 [22].

In 2021, Colombia produced 7.8 billion liters of cow’s milk [23], contributing 12% to the agricultural GDP and creating 700,000 direct jobs across all production stages [24]. Additionally, the apparent annual per capita consumption was 162 L per person [23]. These figures, along with the widespread consumption of dairy products, underscore the essential role of milk in Colombian households.

Livestock activity in the Caribbean region of Colombia is rapidly growing, with an average daily milk production of 113,664 L [25]. Over the past decade, products like meat and milk have begun reaching international markets [26]. However, many milk-producing farms are affected by environmental contamination from open-air dumps, burning of waste, charcoal production, quarrying, industrial discharges and wastewater from populated areas lacking proper sanitation [26]. Additional contamination sources in the livestock sector include the indiscriminate use of agrochemicals, chemical or organic fertilizers and proximity to populated and industrial areas and highways [27]. These factors diminish the quality and safety of dairy products.

This study is primarily motivated by the identification of a deeply rooted cultural belief in the Colombian Caribbean that raw cow’s milk consumption promotes bone health. This perception leads to its widespread and frequent intake, especially among vulnerable groups such as children and the elderly. Despite this widespread practice, there is a notable lack of scientific data on the composition and safety of raw cow’s milk in the region. This research addresses that gap by being the first to evaluate the presence of metals in locally produced milk, an area previously overlooked in the literature. By analyzing metal concentrations, evaluating health risks for both adults and children, and comparing the results with internationally recognized safety standards, this study offers critical insights into the nutritional quality and potential health implications of raw milk consumption in this understudied region.

As milk consumption rises, ensuring its safety is crucial due to the potential contamination by toxic metal(loid)s, which can accumulate in the food chain and impact human health. Quantifying these contaminants in milk provides insight into sanitary quality and environmental pollution in production areas. This study focuses on measuring Pb, Cr, As, and Hg levels in raw cow milk from farms in the Caribbean region of Colombia and.

## Materials and Methods

### Study Area

**Fig. 1:**
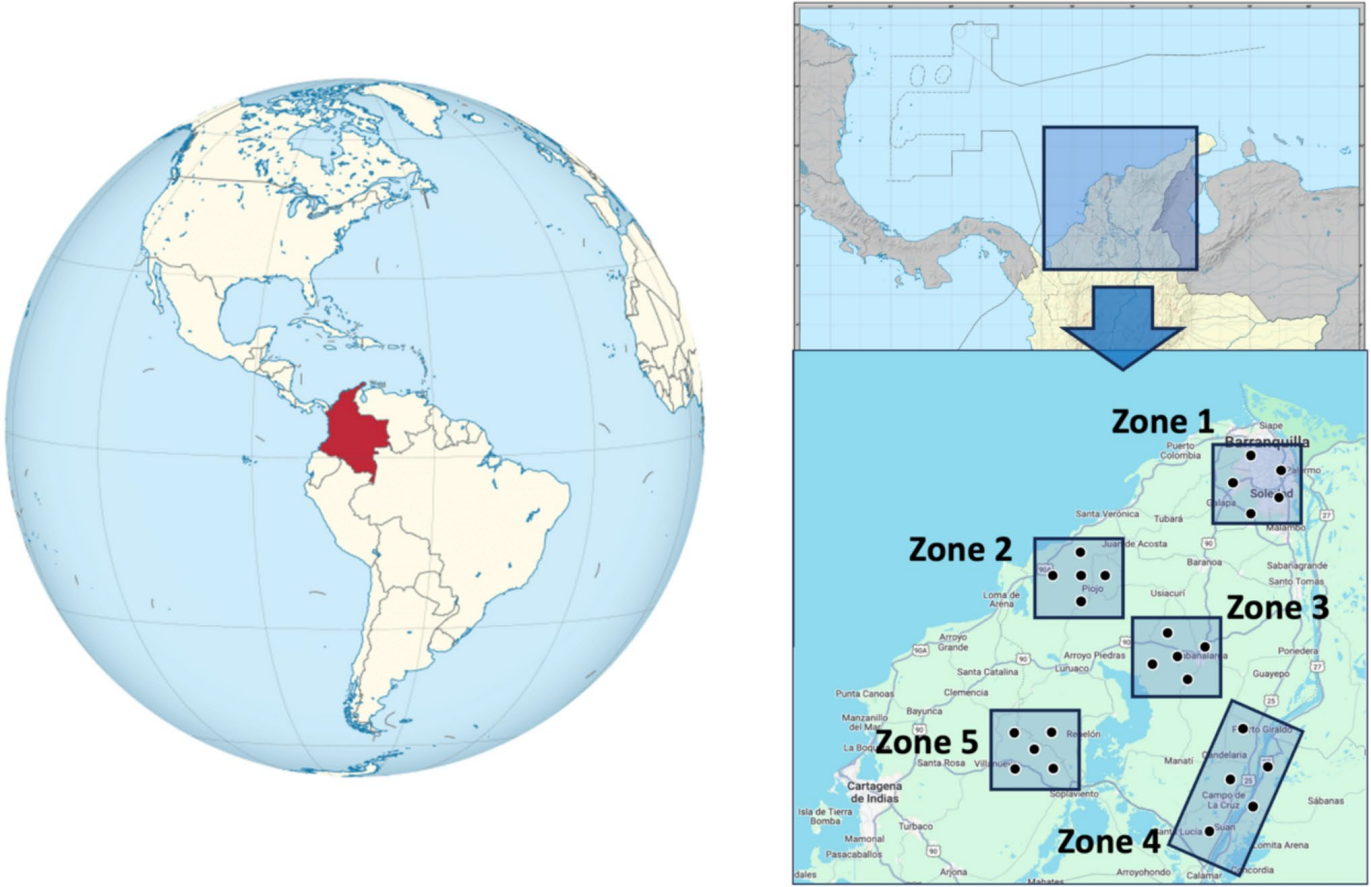
Geographical distribution of milk sampling sites in this study

The Department of Atlántico, located in the Caribbean region of northern Colombia, spans from 10°15′36′′ to 11°06′37′′ north latitude and from 74°42′47′′ to 75°16′34′′ west longitude. It borders the Caribbean Sea to the north and northeast, the Magdalena River to the east, and the Department of Bolívar to the south, southwest, and west [28] (Fig. 1). Covering 3386 km^2^, Atlántico includes 23 municipalities organized into five areas: Metropolitan, Coastal, Central, Eastern and Southern [29].

The department features primarily uniform geographical relief, except for low mountains in the Piojó and Hibácharo ranges. Its vegetation is predominantly tropical dry forest, which has significantly diminished due to agriculture and livestock expansion, leaving only remnants [30]. Along the northern coast, marine-coastal ecosystems such as mangroves, beaches, dunes, and lagoons face erosion, sedimentation, and pollution from industrial discharges [31].

Atlántico’s climate is predominantly dry and warm, with an average annual temperature of about 28 °C and maximum temperatures reaching up to 40 °C. Annual rainfall averages between 500 and 1500 mm [32]. There are two main seasons: a dry season from December to March and a rainy season from April to November [33].

### Sample Collection

Figure 1 shows the five sampling zones where raw cow’s milk samples were collected from 25 cattle farms in the Caribbean region of Colombia. The zones were selected based on their proximity to industrial and urban areas, high-traffic roads, natural reserve areas with little human intervention and areas adjacent to continental water ecosystems, such as the El Guájaro Reservoir and the Magdalena River.

Each farm completed a characterization form to provide the following information: farm code, municipality, georeferencing, types of feed used, use of agrochemicals (such as biofertilizers and pesticides), water supply source, water availability, distance of pastures/forages from the main road, exploitation system, production model and milking system implemented.

Milk samples were collected directly from storage tanks after morning milking, following the protocol by O’Connell et al. [34]. A total of 100 samples, each containing 250 mL, were obtained. Two samples were taken from each farm across different months to capture seasonal variation: one during the dry season (December 2018–February 2019) and the other during the rainy season (September–November 2019). Samples were stored in sterilized bottles at 4 °C until laboratory analysis [35].

### Analysis of Samples

For the analysis of Pb, Cr and As concentrations, separate samples (5 mL in each sample) were digested with HNO_3_/HCl (8/2 v/v) in a microwave oven (in triplicate) using the AOAC 2000 Method. Pb and Cr concentrations were determined using a Thermo ICE 3500 atomic absorption spectrometer with a graphite furnace atomizer module. As concentrations were quantified using atomic fluorescence spectrometry with a hydride generation system (HG-AFS) on a PSA 10.055 Millennium–Excalibur analyzer, following the procedures described in the application notes APP012. Hg concentrations were determined according to the guidelines established by the U.S. Environmental Protection Agency (EPA 7470) using a direct mercury analyzer DMA80 Tricell by Milestone. Analytical method quality control was performed by sample fortification using the standard addition method. Recovery percentages ranged from 88 to 94%, with a CV% below 3.5%. Detection limits for different metal(loid)s were 0.2 μg/L for Cr, 0.5 μg/L for Pb, 0.8 μg/L for As and 0.1 μg/L for Hg. All reagents used were of analytical grade from Merck Millipore. Table S1 provides a summary of the analytical quality control criteria applied in this study.

### Human Health Risk Assessment

A preliminary survey was conducted, targeting residents in farms and surrounding areas from whom raw cow’s milk samples were collected. The survey was randomly administered to 140 individuals from the general population. Participants were asked to complete a questionnaire regarding their milk consumption habits, including the weekly frequency of intake, the total quantity consumed and the different forms in which milk was ingested. The sampled population was divided into two groups: children (< 10 years) and the rest of the population, including men and women.

The health risks associated with metal(loid)s was estimated through the evaluation of non-carcinogenic and carcinogenic risk. The former was assessed by calculating the estimated daily intake (EDI) (µg/kg/day) using the following equation [36]:

1 $$\mathrm{EDI}=\frac{{C}_{m} X\;DI}{BW}$$

where *C*_*m*_ is the 75th percentile value concentration of metal(loid)s in raw milk (µg/L), DI is the daily milk intake (L/day) and BW is the average body weight of the participants (kg).

The risk characterization was performed by calculating the hazard quotient (HQ), which relates the estimated daily intake of a chemical substance to a reference dose (RfD µg/kg/day) defined as the maximum tolerable daily intake of a metal(loid)s that does not cause adverse health effects.

2 $$\mathrm{HQ}=\frac{EDI}{RfD}$$

When the HQ value is less than 1, it is unlikely that adverse effects will occur, and the risk can be considered insignificant. However, if HQ is greater than 1, it indicates that the EDI of a specific metal(loid) exceeds the RfD, suggesting the presence of potential risk associated with that metal(loid) [37]. The RfD values for Cr, As and Hg were 3, 0.3 and 0.3 µg/kg/day, respectively [38–40]. In 2010, the RfD value of 3.57 µg/kg/day for Pb determined by the WHO (1986) was withdrawn by EFSA (2010) and replaced with three Benchmark Dose Lower Confidence Limits (BMDLs) to assess the effects of lead exposure in humans: BMDL10 for nephrotoxicity at 0.63 µg/kg bw/day, BMDL01 for cardiovascular effects at 1.5 µg/kg bw/day and BMDL01 for developmental neurotoxicity.

The hazard index (HI) was used to assess the potential risk to human health from ingesting various metal(loid)s. The HI was calculated by summing the individual HQs of the elements in each population group [41].

3 $$\mathrm{HI}=\sum \mathrm{HQ}$$

An HI value greater than 1 indicates an unacceptable risk of non-carcinogenic health effects, while an HI of less than 1 indicates that the risk is acceptable for human health [42].

The probability of a person developing cancer over their lifetime as a result of exposure to contaminated milk consumption was estimated using the carcinogenic risk (CR). The CR for Pb, Cr and As were calculated using Eq. 4:

4 $$CR=\frac{C_m\;\times\;EF\;\times\;ED\;\times\;IR\;\times\;0.001}{TA\;\times\;BW}\times\;CSF$$

where *C*_*m*_ is the average concentration of metal(loid)s in milk (µg/L); EF is the exposure frequency (350 days/year); ED is the exposure duration, for adults (30 years) and children (6 years); IR represents the ingestion rate IR represents the ingestion rate as determined by the surveys (0.95 kg/day for children and 1.25 kg/day for adults); AT is the averaging time (day): for carcinogens 70 (lifetime) × 365 days; BW is the average body weight of participants (30 kg for children and 70 kg for adults); and CSF corresponds to the carcinogenic risk slope factor for As, Cr and Pb were 1.5, 0.5 and 0.0085 mg/kg/day, respectively [43]. For this scenario, we assumed the adult was exposed 7 days per week, 52 weeks per year, for a total of 70 years, and the children were exposed for 10 years.

The total carcinogenic risk (TCR) was obtained by summing the CRs of the elements, assuming their effects are additive. A CR of 10^−6^, representing a probability of 1 in 1,000,000 people, was considered acceptable [44, 45].

Following USEPA guidance, a CR value lower than 10^−6^ represents negligible levels, values 10^−6^ < CR < 10^−4^ are acceptable levels, while CR > 10^−4^ signifies a high cancer risk to humans [46].

According to European Union regulations (European Commission Regulation (EU) 2023/915), 0.0200 mg L^−1^ is defined as the maximum permitted amount of Pb in milk. The current findings demonstrated that the Pb levels in milk collected from both dairy farms and collection centers were above the permissible level.

### Statistical Analysis

The results of the milk sample analysis are presented as mean ± standard deviation. The application of the Kolmogorov test showed a departure from a normal distribution, signifying non-normality in the data. The Kruskal–Wallis test was employed to compare the concentrations of metal(loids)s in milk among different sampling zones. Significant relationships between potentially toxic elements and their common origins were determined through principal component analysis. All statistical analyses were conducted using R Studio version 4.1.2, with a significance level set at *p* ≤ 0.001 and *p* ≤ 0.05.

## Results and Discussion

### Concentrations of Metal(loid)s in Raw Milk

Table 1 shows the concentrations of metal(loid)s in raw cow’s milk samples collected from different zones in the Caribbean region during the two climatic seasons. Generally, the concentrations of metal(loid)s followed this decreasing order: Cr > Pb > As > Hg.

**Table 1:** Mean and median concentrations and interquartile range (IQR) of metal(loid)s (µg/L) in raw cow's milk samples collected in the Caribbean region

Zones	Pb	Cr	As	Hg
1	5.41, 3.73 (7.77)	117, 91 (243)	2.82, 2.83 (1.58)	0.64, 0.64 (0.86)
2	2.65, 1.39 (5.51)	50, 35 (91)	2.43, 2.47 (3.42)	0.08, 0.05 (0.06)
3	2.57, 2.28 (3.25)	108, 88 (236)	2.45, 2.90 (3.42)	0.23, 0.05 (0.10)
4	2.72, 2.23 (4.24)	82, 37 (91)	2.41, 3.16 (3.26)	0.27, 0.26 (0.38)
5	2.62, 2.21 (4.50)	47, 26 (75)	2.20, 2.33 (2.33)	0.57, 0.27 (0.85)
Total	3.19, 2.23 (4.78) 0.25-5.03^a^	81, 44 (128) 2.82-131^a^	2.46, 2.93 (2.38) 1.19-3.56^a^	0.35, 0.18 (0.41) 0.05-0.46^a^

^a^IQR (25^th^ percentile-75^th^ percentile)

**Fig. 2:**
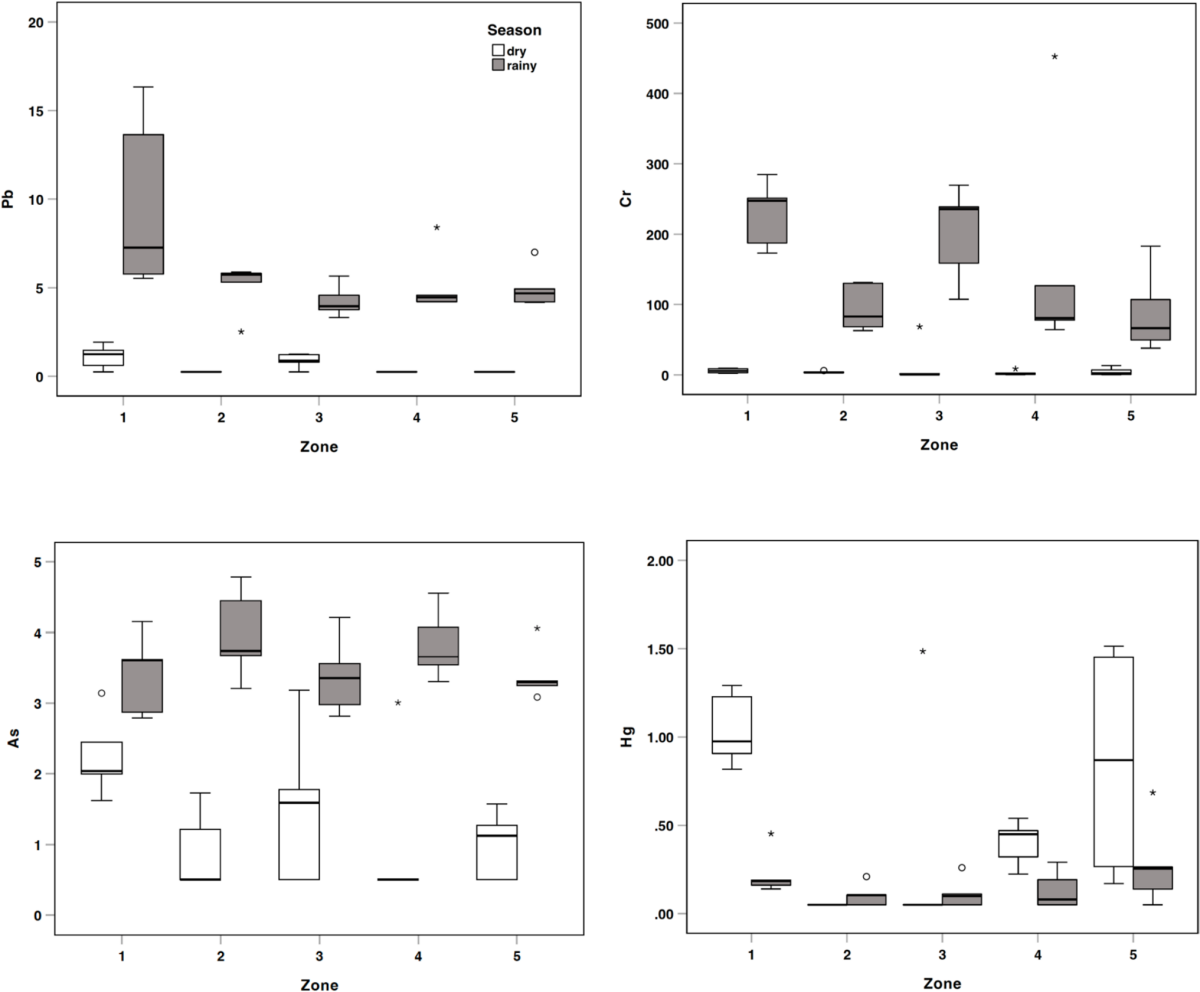
Concentrations of Pb, Cr, As and Hg in milk by zone. Boxes depict 25th, 50th and 75th percentiles and whiskers minimum and maximum values. Outliers are represented by open circles and extremes by asterisk

Regarding the concentrations of metal(loid)s in the two climatic seasons (Fig. 2), it was found that Cr exhibited the highest concentrations, while Hg showed the lowest. During the dry season, As concentrations were higher than those of Pb, whereas in the wet season, Pb became the second most abundant element. The concentrations of Pb, Cr and As were significantly higher during the rainy season compared to the dry season (*p* < 0.001). In contrast, Hg levels were elevated in the dry season, although this difference was not statistically significant. This seasonal pattern may be attributed to the reduced rainfall during the dry period, which limits surface runoff and leaching, potentially leading to the accumulation or retention of Hg in the upper soil layers. The environmental mobility and partitioning of Hg are strongly influenced by its chemical speciation and interactions with various soil constituents, including organic matter, inorganic minerals, biota, pH and redox conditions [47]. Furthermore, higher temperatures during the dry season may enhance microbial activity in soils, potentially increasing mercury methylation rates and thereby raising its bioavailability in biological systems [48].

The higher concentrations of other metal(loid)s observed during the rainy season can be attributed to several factors: (i) rainfall can leach metal(loid)s from the soil, transporting them into deeper soil layers and water bodies, which increases metal(loid) concentrations in these environments [49], (ii) intense precipitation can cause soil runoff, carrying particles containing metal(loid)s into aquatic ecosystems [50], (iii) in certain areas, agricultural activities often coincide with the rainy season, leading to the release of metal(loid)s into the environment [51]; (iv) rainfall can alter soil pH by leaching basic cations, increasing soil acidity, which in turn promotes the dissolution of metal(loid)s in soil water and enhances their availability to plants and entry into food chains [52], and (v) seasonal flooding can displace substantial amounts of soil and sediment, releasing and transporting metal(loid)s to new areas [53]. Overall, it is important to note that seasonal or climatic variations alone are generally not decisive factors in determining metal(loid) concentrations in milk. Other factors, such as soil quality, exposure to contamination sources, animal feed and regional agricultural practices [54], play a more significant role in influencing these concentrations.

The concentrations obtained across different sampling zones were not significantly different, except for Hg, where significant differences were observed between Zone 1 and 2, Zone 1 and 3 and Zone 2 and 5. Regardless of the season, whether dry or rainy, Zone 1 consistently exhibited the highest concentrations of all metal(loid)s. The mean Pb concentration during the rainy season (9.71 µg/L) was notably around nine times higher than that observed during the dry season (1.10 µg/L). Additionally, the highest mean Cr concentration (228.81 µg/L) was recorded in Zone 1 during the rainy season, while Zone 1, which includes both the metropolitan area of Barranquilla and the municipality of Tubará, is marked by rapid population growth, urban expansion into rural areas [55], deforestation and significant landscape degradation [56]. Elevated metal(loid)s concentrations in this area are likely linked to various industrial activities, including the production of clay blocks, cement, ceramics, textiles, leather tanning, metalworks and pesticide manufacturing, as well as waste incineration and disposal operations [57, 58]. Together, these factors create an environment where metal(loid)s are widely distributed across air, soil, and water. Cows exposed to these contaminants may accumulate metal(loid)s, which are subsequently detected in raw milk.

In contrast, Zone 2, located in the rural area of the municipality of Piojó, is less influenced by human, industrial and vehicular traffic activities. This zone recorded the highest mean As concentration, reaching 3.97 µg/L. During the sample collection, it was observed that farmers applied various agricultural inputs without proper dosing and in a low-tech manner. Several studies have shown that As contamination in agricultural soils is often linked to the application of pesticides and phosphate fertilizers [59, 60]. Additionally, it is possible that natural As deposits in the local geology could contribute to contamination, as they may seep into water and soil systems, ultimately impacting the broader ecosystem [61]. On the other hand, the average concentrations of metal(loid)s analysed in the raw cow milk samples for this study were compared with those recorded in recent research (2014–2024) conducted in different countries (Table 2). All concentrations measured in this study were lower than those previously reported in Colombia [62, 63] and comparable to those reported in countries such as Bangladesh, Sri Lanka, India and Peru [64–67]. Quartile analysis revealed that the average Pb concentration in these milk samples exceeded 25% of global Pb levels reported in milk but remained below the 50th percentile. This suggests that, while Pb levels here are not extremely high, they still surpass a notable portion of worldwide observations in China and Ethiopia [16, 17, 68, 69]. The average Cr concentration fell within the third quartile of global data, indicating that its content in milk is higher than approximately 50% of worldwide observations [11, 15, 16, 21, 46, 69, 70]. In contrast, the average concentrations of Hg and As were notably lower, falling below the 25th percentile of global data. It is important to highlight that the concentrations of Pb, Cr, Hg and As in all milk samples remained below the permissible limits established for these elements, according to the guidelines provided by MHSP [71], EU [72, 73], JOINT FAO/WHO Codex Alimentarius [74], USDA [75] and summarized in Table 2. This suggests that this food does not pose an immediate health threat. However, it is crucial to consider that with high consumption, there could be a potential risk. These potentially toxic elements do not have a physiological function in the body and can accumulate over time, potentially leading to chronic illnesses and possible harm to the population. Therefore, it is imperative to assess the risk they pose to human health.

**Table 2:** Concentrations of Pb, Cr, Hg, and As in raw cow milk and their comparison with other studies. The concentrations are expressed in µg/L

Region	Anthropogenic activities	Pb	Cr	Hg	As	Reference
Colombia	Mining, use of pesticides and biosolids	150±23		120±4	277±49	Londoño [62]
Iran	Industries			7.29-14.95	15.20-25.90	Arianejad et al. [76]
Bangladesh	-	15±80	373±80			Muhib et al. [66]
Colombia	-	5.88				Serna and Valderrama [63]
Guangxi province, China	-	1.46±2	12.52	5.20	0.86	Zhou et al. [69]
China	Mining and industrial activities	8.50±7.42	34.58±24.15	2.31±1.40	1.35±0.32	Qu et al. [46]
Mexico	Industrial effluents discharge	30.99±10.33	30.99±20.66		123.96±82.64	Castro-González et al. [11]
Romania	Mining	24.79±15.49				Miclean et al. [45]
China	Industry, mining, and smelting	1.75±3.73			0.31±1.02	Zhou et al. [17]
Tangshan, China	Industry (steel, cement, and waste incineration)	1.22±1.62	0.87±1.02		0.06±0.20	Zhou et al. [21]
Shandong province, China	Intensive agriculture	24.17±14.25	15.49±9.50		4.76±2.27	Chen et al. [77]
Sri Lanka	Pesticide and transhumance from motorways	15.4±5.15	214±190		13.1±2.92	Diyabalanage et al. [65]
Ecuador	Steel industry and vehicular traffic	214.86		0.092	0.030	De La Cueva et al. [78]
India	Intensive agriculture, animal husbandry, industries	200±30	530±100			Yasotha et al. [67]
Ladakh, India	Construction and tourist	5.6±0.3			5.2±0.3	Giri et al. [79]
China	Industry (cement and power plants)	2.25±3.26	2.35±2.07		0.43±0.41	Su et al. [15]
China	Extensive agricultural and industrial activities	2.86±0.96	1.21±1.58		0.43±0.21	Su et al. [16]
Italy	Industrial effluents, sewage discharge, chemical fertilizer	20.66±61.98	10.33±10.33		0.00±0.00	Monteverde et al. [70]
Turkey	-	0.87			1.04	Özbay et al. [80]
Peru	Industry (metallurgical and cement), traffic, agriculture.	29.95±22.72			10.33±4.13	Chirinos-Peinado et al. [64]
Brazil	Agriculture and industrial effluents.	43±2				Oliveira et al. [81]
Ethiopia		2310	369			Tola et al. [68]
Colombia		3.19±1.77	80.69±55.86	0.35±0.34	2.45±0.72	This study
Permissible limit		20				MHSP [71]
		20				EU [72, 73]
		20				JOINT FAO/WHO Codex Alimentarios [74]
		50	300	10	100	USDA [75]

*MHSP*: Ministry of Health and Social Protection

### Distribution and Correlation of Metal(loid)s in Raw Milk Across Study Zones

**Fig. 3:**
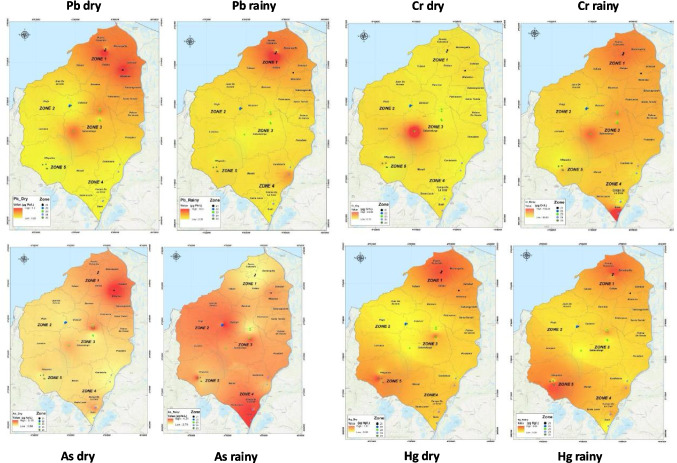
Spatial distribution of metal(loid)s in milk samples collected from 5 zones in the Department of Atlántico during both climatic periods

Figure 3 illustrates the spatial distribution of metal(loid)s, with darker tones indicating areas of higher contamination. The results reveal similar distribution patterns across seasons, with Zones 1 and 3 in the northern and central Colombian Caribbean exhibiting the highest concentrations of Pb, Cr and Hg. These zones are influenced by high-traffic roads, potentially elevating Pb levels from vehicle exhaust [67]. Industrial activity and urban density likely contribute to higher metal(loid)s contamination in Zone 1 [82, 83]. Additionally, Zone 5 in the east, near El Guájaro Reservoir, shows elevated Hg levels in both dry and rainy seasons, likely due to upstream Hg contamination in the Magdalena River [84, 85]. In contrast, Zone 2, a rural area distant from industrial sites, has the lowest metal(loid contamination, aligning with findings that rural areas generally show lower metal(loid) levels in milk due to minimal vehicular and industrial influence [15, 86].

Principal component analysis (PCA) was used to examine metal(loid) relationships across the five sampling zones, as shown in Fig. 3. In the dry season, PCA reduced the data to two main components that account for 69% of the total variation, with As, Pb and Cr showing strong loadings (Fig. 3a). During the rainy season, PCA explained 64.8% of variation, highlighting Pb, Cr and Hg as significant contributors (Fig. 3b).

In the dry season, As and Pb concentrations were correlated and primarily associated with Zones 1 and 3, where contamination may result from proximity to roads, water sources (streams, groundwater, reservoirs) and agricultural practices involving biofertilizers and pesticides. Zone 1, in particular, is close to industrial facilities producing construction materials, metal products, batteries and waste management services [57], while Zone 3’s farms are within 1 km of the heavily trafficked highway connecting Barranquilla and Cartagena.

In the rainy season, Pb and Cr levels were linked to Zones 1, 3 and 4, where grazing and water sources play key roles; Zone 4, for example, relies on the Magdalena River, which is known to carry heavy metal(loid) contaminants [84]. As levels correlated with Zones 3 and 4, where biofertilizers are commonly used, and some farms rely on groundwater. Hg concentrations were primarily associated with Zone 5, which draws water from the Repelón irrigation district and is influenced by El Guájaro Reservoir, a known site of Hg contamination in water and surrounding soils [51, 85, 87]. The presence of Hg likely impacts agricultural and livestock activities in this area.

### Potential Health Risks Evaluation

#### Non-Carcinogenic Risk and Carcinogenic Risk

Based on the information collected from the survey applied in the study’s influence zone, non-cancerous and cancerous risks associated with each metal(loid)s were evaluated. These values were calculated assuming an average body weight of 70 kg for adults and 30 kg for children, with a daily milk consumption of 1.25 L and 0.95 L, respectively.

The values for estimated daily intake, hazard quotient, hazard index, carcinogenic risk, and total carcinogenic risk are presented in Table 3.

**Table 3:** Estimation of potential non-cancerous and cancerous risks in the population, due to consumption of raw cow milk contaminated with potentially toxic elements

	Children			Adults		
	EDI	HQ	CR	EDI	HQ	CR
Pb*	0.159	0.253	1.1 10^−7^	0.090	0.142	3.1 10^−7^
Pb**	0.159	0.106		0.090	0.060	
Cr	4.131	1.377	1.7 10^−4^	2.330	0.777	4.8 10^−4^
As	0.113	0.377	1.4 10^−5^	0.064	0.213	3.9 10^−5^
Hg	0.014	0.048		0.008	0.027	
HI		2.161			1.219	
TCR			1.8 10^−4^			5.2 10^−4^

Pb*: BMDL10 for nephrotoxicity at 0.63 µg/kg bw/day. Pb**: BMDL01 for cardiovascular effects at 1.5 µg/kg bw/day

The highest values of EDI values were observed for Cr, while Hg had the lowest values. Intake levels followed the same decreasing order for both adults and children: Cr > Pb > As > Hg. In children, these intake values were nearly twice as high as those for adults across all four elements. Importantly, none of the EDI values exceeded the RfD limits set by JECFA and USEPA, indicating no immediate health threat.

**Fig. 4:**
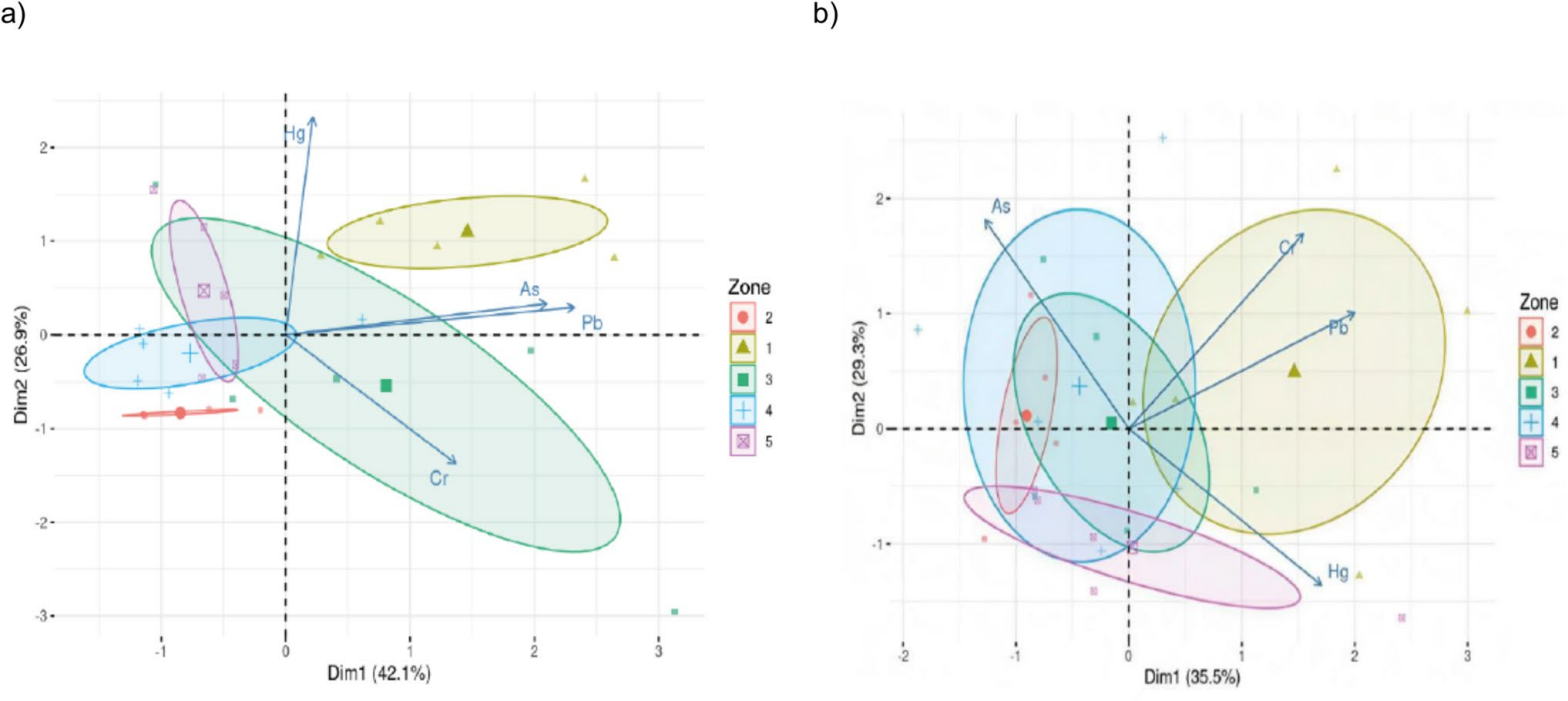
Principal component analysis of concentrations of metal(loid)s in milk and variables during the dry (**a**) and rainy (**b**) period

The HQ assessment for milk consumption (Fig. 4) showed that HQ values for all metal(loid)s were below 1 in both population groups, except for Cr in children (HQ = 1.377). This indicates a high risk of significant adverse health effects under the current conditions. Additionally, the HQ for Cr in adults (HQ = 0.777) was nearing the threshold of 1, suggesting that exposure to this metal is approaching the reference limit and requires attention due to its potential toxic effects.

The HI was slightly above the safety threshold (< 1) for adults (HI = 1.219). The HI slightly exceeded the safety threshold (< 1) for adults (HI = 1.219). However, for children, it was significantly higher, nearly double this threshold (HI = 2.161), with Cr being the primary contributor to this elevated risk (HQ = 1.377) (Fig. 4). This finding highlights a potential health risk for children from milk consumption, as their developing digestive systems are more efficient at absorbing toxic metal(loid)s [88]. Similar trends, with HI values exceeding 1 in children and lower values in adults, have been reported in studies on milk consumption in Peru [64]. Furthermore, the HI values observed in this study are higher than those documented for cow’s milk consumption in Iran [89] and Romania [45].

**Fig. 5:**
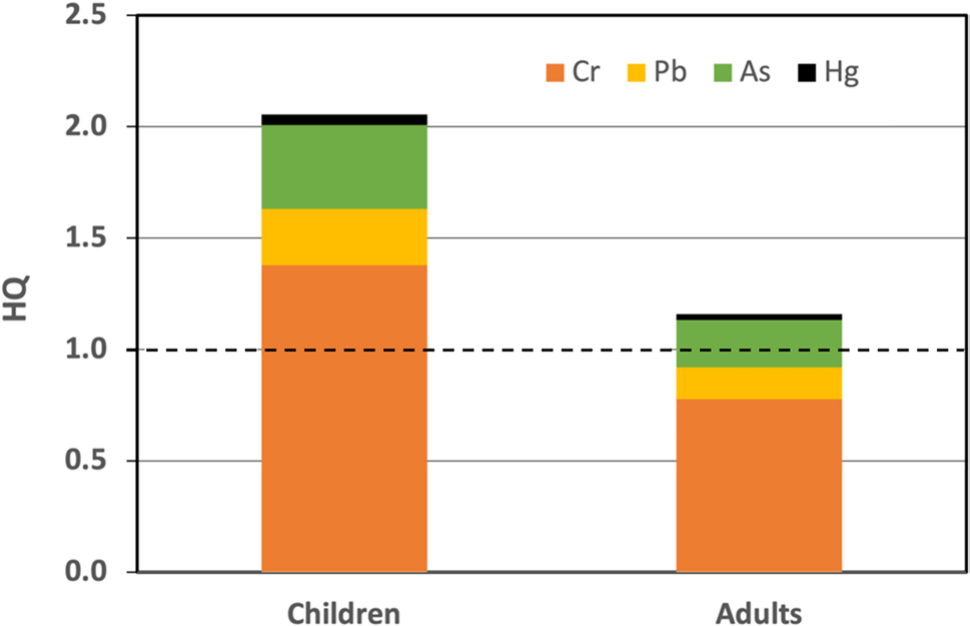
Values of hazard quotients (HQ) for Pb, Cr, As and Hg in adults and children. Dashed dotted line is the reference limit. Values above 1 suggest the presence of potential risk associated with that metal

The elevated HI in children could result in both short- and long-term health effects, depending on exposure duration and the toxicity of the specific metal(loid) involved. To mitigate these risks, it is essential to reduce metal(loid) exposure through improved milk production practices and by enforcing stricter limits on allowable metal(loid) concentrations in food, safeguarding children’s health (Fig. 5).

The estimated cancer risk (CR) values for Pb and As in both population groups were below the incremental probability of a person developing cancer at some point in a lifetime (CR = 10⁻⁶), indicating a negligible cancer risk. In contrast, CR values for Cr exceeded the acceptable threshold, being twice as high for children and five times higher for adults, with values ranging from 1.7·10⁻^4^ to 4.8·10⁻^4^, respectively (Table 3). Total CR for all elements surpass the USEPA guideline across all age groups, with adults (TCR = 5.2·10⁻^4^) facing the highest cancer risk.

The CR values for As and Pb were similar to those reported in studies on cow’s milk consumption in India (As: 1.47·10^−5^, Pb: 9.5·10^−8^) [79], but lower for Pb in Bangladesh (3.5·10^−5^) [66], where the cancer risk was deemed negligible. The total cancer risk (TCR) value for both populations was slightly above the acceptable limits due to greater Cr values (Table 3).

Chromium, found naturally and introduced through industrial activities, exposes the population mainly via food, water, and air. Both trivalent (Cr(III)) and hexavalent (Cr(VI)) chromium forms can be present, with Cr(VI) being a potent carcinogen linked to lung cancer and more toxic than Cr(III) [90]. Chromium can cross the placental barrier, posing fetal risks, including embryotoxicity [91], fetotoxicity [37], and developmental malformations. Individuals near contaminated areas face increased risks of congenital malformations, low birth weight, and premature birth [92].

## Conclusions

The results of this study indicate that raw cow milk produced in the Colombian Caribbean region contains metal(loid)s, with the highest concentrations of Pb, Cr and As recorded during the rainy season and the highest Hg concentrations during the dry season. Chromium consistently showed the highest concentrations across all samples in both seasons. Geographically, Zone 1 exhibited the highest contamination levels for all elements. Although metal(loid) concentrations were below permissible limits, the Hazard Index (HI) for children slightly exceeded 1, suggesting a notable health risk from milk consumption for this group. There was no public health concern for cancer risk from Pb and As exposure through cow’s milk consumption, as levels were below the USEPA threshold (10⁻⁶) across all age groups. However, the estimated cancer risk for Cr exceeded this threshold, indicating a potential public health concern, particularly for children, who were found to have the highest cancer risk. Given the toxic potential of metal(loid)s and milk’s high consumption rate, regular monitoring of these contaminants in milk and other environmental sources is essential. Preventive actions to regulate raw milk consumption and limit contaminant exposure are recommended to protect human health.

## Supplementary Information

Below is the link to the electronic supplementary material.

ESM1 (DOCX 14.4 KB)

## Data Availability

The datasets generated during and/or analysed during the current study are not publicly available but are available from the corresponding author on request.
