# Supplementary material for: Health Risk Assessment of Metal(loid) Contamination in Raw Cow’s Milk from the Colombian Caribbean Region
Source: Biol Trace Elem Res. 2025 Aug 7;204(2):1043–56. doi: 10.1007/s12011-025-04728-5 (PMC12847161; doi:10.1007/s12011-025-04728-5)
Supplement: Supplementary file 1 — (DOCX 14.4 KB) [file 12011_2025_4728_MOESM1_ESM.docx]

**SUPPLEMENTARY MATERIAL FOR THE PAPER**

**Health Risk Assessment of Metal(loid) Contamination in Raw Cow’s Milk from the Colombian Caribbean Region**

Fabio Fuentes-Gandara^a,*^, Jaime Barreto-Cañas^b^, Siday Marrugo-Madrid^c^ José Marrugo-Negrete^c,*^ José Pinedo-Hernández^c,*^, Sergi Díez^d,*^

^a^ Universidad de la Costa, Department of Natural and Exact Sciences, Barranquilla, Colombia

^b^ Universidad del Atlántico, Faculty of Basic Sciences, Barranquilla, Colombia

^c^ University of Córdoba, Faculty of Basic Sciences, Department of Chemistry, Water, Applied and

Environmental Chemistry Group, Monteria, Colombia

^d^ Environmental Chemistry Department, Institute of Environmental Assessment and Water Research, IDAEA-CSIC, E-08034, Barcelona, Spain

**Table S1.** Analytical Quality Control Parameters

| **Analyzer** | **Metal(loid)** | **DL (µg/L)** | | **LR** | **Units** | **r** | **Spiked (µg/L)** | | **% R** | **%CV** |
| --- | --- | --- | --- | --- | --- | --- | --- | --- | --- | --- |
| GF-AAS | Cr | 0.2 | | 0.5-5.0 | µg/L | 0.9996 | 2.5 | | 92.1 | 2.8 |
|  | Pb | 0.5 | | 0.5-10 |  | 1.0000 | 5 | | 88.0 | 3.2 |
| HG-AAS | As | 0.8 | | 0.8-30 |  | 0.9985 | 15 | | 94.0 | 2.3 |
| DMA-80 | Hg | 0.1 | | 0.5-4.5 | ng | 0.9997 | 10 | | 93.5 | 2.2 |
|  |  |  |  | 4.5-20 |  | 0.9996 |  |  |  |  |
|  |  | |  | 20-600 |  | 0.9991 |  |  | |  |
|  |  |  |  | 4.5-20 |  | 0.9996 |  |  |  |  |
|  |  |  |  | 20-600 |  | 0.9991 |  |  |  |  |

DL: detection limit; LR: Linearity range
